# Evaluating a Web-Based Social Anxiety Intervention Among University Students: Randomized Controlled Trial

**DOI:** 10.2196/jmir.8630

**Published:** 2018-03-21

**Authors:** Hugh Cameron McCall, Chris G Richardson, Fjola Dogg Helgadottir, Frances S Chen

**Affiliations:** ^1^ Department of Psychology University of British Columbia Vancouver, BC Canada; ^2^ School of Population and Public Health University of British Columbia Vancouver, BC Canada; ^3^ Vancouver CBT Centre Vancouver, BC Canada

**Keywords:** social anxiety, clinical trial, internet, cognitive behavior therapy

## Abstract

**Background:**

Treatment rates for social anxiety, a prevalent and potentially debilitating condition, remain among the lowest of all major mental disorders today. Although computer-delivered interventions are well poised to surmount key barriers to the treatment of social anxiety, most are only marginally effective when delivered as stand-alone treatments. A new, Web-based cognitive behavioral therapy (CBT) intervention called Overcome Social Anxiety was recently created to address the limitations of prior computer-delivered interventions. Users of Overcome Social Anxiety are self-directed through various CBT modules incorporating cognitive restructuring and behavioral experiments. The intervention is personalized to each user’s symptoms, and automatic email reminders and time limits are used to encourage adherence.

**Objective:**

The purpose of this study was to conduct a randomized controlled trial to investigate the effectiveness of Overcome Social Anxiety in reducing social anxiety symptoms in a nonclinical sample of university students. As a secondary aim, we also investigated whether Overcome Social Anxiety would increase life satisfaction in this sample.

**Methods:**

Following eligibility screening, participants were randomly assigned to a treatment condition or a wait-list control condition. Only those assigned to the treatment condition were given access to Overcome Social Anxiety; they were asked to complete the program within 4 months. The social interaction anxiety scale (SIAS), the fear of negative evaluation scale (FNE), and the quality of life enjoyment and satisfaction questionnaire—short form (Q-LES-Q-SF) were administered to participants from both conditions during baseline and 4-month follow-up lab visits.

**Results:**

Over the course of the study, participants assigned to the treatment condition experienced a significant reduction in social anxiety (SIAS: *P*<.001, Cohen *d*=0.72; FNE: *P*<.001, Cohen *d*=0.82), whereas those assigned to the control condition did not (SIAS: *P*=.13, Cohen d=0.26; FNE: *P*=.40, Cohen *d*=0.14). Additionally, a direct comparison of the average change in social anxiety in the 2 conditions over the course of the study showed that those assigned to the treatment condition experienced significantly more improvement than those assigned to the control condition (SIAS: *P*=.03, Cohen *d*=0.56; FNE: *P*=.001, Cohen *d*=0.97). Although participants assigned to the treatment condition experienced a slight increase in life satisfaction, as measured by Q-LES-Q-SF scores, and those assigned to the control condition experienced a slight decrease, these changes were not statistically significant (treatment: *P*=.35, Cohen *d*=−0.18; control: *P*=.30, Cohen *d*=0.18).

**Conclusions:**

Our findings indicate that Overcome Social Anxiety is an effective intervention for treating symptoms of social anxiety and that it may have further utility in serving as a model for the development of new interventions. Additionally, our findings provide evidence that contemporary Web-based interventions can be sophisticated enough to benefit users even when delivered as stand-alone treatments, suggesting that further opportunities likely exist for the development of other Web-based mental health interventions.

**Trial Registration:**

ClinicalTrials.gov NCT02792127; https://clinicaltrials.gov/ct2/show/record/NCT02792127 (Archived by WebCite at http://www.webcitation.org/6xGSRh7MG)

## Introduction

### Background

Social anxiety disorder is one of the most common anxiety disorders, with approximately 13% of people being affected at some point in their lives [1]. Even people who are below the threshold for clinical diagnosis experience substantial distress and functional impairment [2]. Furthermore, research has shown that social anxiety symptoms tend to be persistent at all levels of severity [3]; that social anxiety is closely related to disorders such as substance abuse, disordered eating, and mood disorders [4]; and that the impacts of social anxiety can be severe in both private and professional domains of life when it is left untreated [5-7]. The effectiveness of various psychotherapeutic and pharmaceutical approaches to treating social anxiety—for example, cognitive behavioral therapy (CBT), selective serotonin reuptake inhibitors—is well documented [8,9]; yet, rates of treatment for social anxiety are some of the lowest among all major mental disorders [10], highlighting the need for the development of more accessible treatment options for social anxiety disorder and subclinical social anxiety alike.

Computer-delivered therapy, including computerized CBT, has become increasingly popular in recent years and holds substantial promise for increasing access to effective treatment options for both depression and social anxiety [11]. One of its major advantages lies in its accessibility to individuals who experience geographic, financial, or personal challenges for human-delivered therapy. Notably, because financial and confidentiality concerns are especially common barriers to treatment for people suffering from social anxiety [10], the privacy and relative affordability of computer-delivered therapy may have particular practical utility for treating social anxiety relative to other mental disorders. Moreover, because students infrequently seek help from professionals for mental health–related problems, but tend to be very comfortable with modern digital technologies, computer-delivered therapies may be especially effective among student populations [12]. Given the high rates of anxiety found among young adults [13], and university students in particular [14], exploring the effectiveness of computer-delivered therapies among student populations may be an especially important area of research.

Computer-delivered therapy programs are not a novel innovation; in fact, they date back to the 1960s [15]. However, a meta-analysis found that the effectiveness of Web-based CBT treatment programs that are not supplemented by human-delivered therapy is minimal [16], suggesting that such programs require improvement before they are delivered as stand-alone treatments. A total of 5 common limitations of many Web-based CBT treatments have been identified [15]. First, many treatments do not offer users individualized programs to address their unique symptoms. Second, many programs tend to provide little visual or audio surrogate human contact, despite research attesting to the importance of therapist-client interaction to a program’s success [17]. Third, many Web-based CBT treatments lack mechanisms to facilitate adherence, and completion rates of programs can be as low as 1% [18]. Fourth, programs often do not provide corrective feedback to participants who misunderstand important aspects of the CBT process, such as designing behavioral experiments or differentiating between thoughts, emotions, and feelings. Finally, although administration of an appropriate dose of treatment is important to CBT’s success (eg, sufficient repetition of CBT exercises) [19], many treatments fail to provide a sufficient dose of treatment to deliver lasting benefits to users.

### Overcome Social Anxiety

A Web-based CBT program designed to reduce social anxiety symptoms among stuttering populations, developed specifically to address the aforementioned 5 limitations, has shown promising preliminary results across 3 evaluative studies [20-22]. Originally called CBTpsych, the program has recently been developed into Overcome Social Anxiety, which is no longer tailored specifically toward stuttering populations. Before this study, Overcome Social Anxiety has not received empirical evaluation. The program’s clinical content consists of 7 modules, as shown in Textbox 1, which are intended to be completed over a 4- to 6-month period. It was created by 2 professional clinical psychologists, and employs established CBT procedures for treating social anxiety. Although participants in this study were given free access to Overcome Social Anxiety, it is also available to the public for purchase. A screenshot of the program is shown in Multimedia Appendix 1.

Overcome Social Anxiety has built-in mechanisms to address each of the 5 common limitations of Web-based CBT treatments identified above. First, it individualizes treatment programs as a function of participants’ responses to questionnaires about the symptoms of social anxiety that they experience. Second, the program provides users with example responses to help ensure that they understand various aspects of the CBT process (eg, how to design effective behavioral experiments), mitigating the need for corrective feedback. Third, to help improve adherence, users are given a limit of 6 months to complete the program and are sent automated email reminders to keep using the program after periods of inactivity. Fourth, the program employs voice recordings of 2 clinical psychologists explaining important aspects of CBT to users, in an effort to more closely mirror psychologist-delivered CBT. Finally, the program is designed to administer a sufficient dose of individualized therapy to effect lasting reductions in users’ social anxiety. Specifically, Overcome Social Anxiety employs all aspects of CBT widely accepted today, including the identification of unhelpful thoughts and avoidance behaviors, psychoeducation on emotions and cognitive errors and unhelpful behaviors, the construction of individual models of social anxiety, the employment of cognitive restructuring strategies, and engagement in exposure exercises in the form of behavioral experiments. For more detailed information about the program, please refer to the studies by Helgadóttir et al [20,21].

### Hypotheses

To investigate the effectiveness of Overcome Social Anxiety, we conducted a randomized controlled trial in a population of university students who reported symptoms of social anxiety but had not received a clinical diagnosis for it. Our primary hypothesis was that participants who were given access to Overcome Social Anxiety would experience a greater decrease in social anxiety symptoms over a 4-month period than those assigned to a wait-list control condition.

**Outline of modules employed in Overcome Social Anxiety.**

Prequestionnaires

- The questions asked in this section are retrieved from file audit data from cognitive behavioral therapy clinical practice. The user is presented with a list of 37 common social anxiety thoughts (eg, “I can’t speak to authority figures”) and a list of 26 common avoidance behaviors (eg, verbal presentations). The user ranks how relevant the thoughts and behaviors are to his or her particular symptoms of social anxiety, which the program then uses to individualize the user’s course of treatment.

Module 1: Thinking exercises

- The virtual therapeutic relationship is established when the real clinical psychologists introduce themselves via a photograph and a prerecorded sound clip. This section is designed to familiarize the user with the program’s methods, such as learning to use feedback via sample answers and voice-overs. Common cognitive errors are described, with exercises designed to educate the user on the relationship between cognition, behaviors, and emotion.

Module 2: Challenging your thinking

- The user is presented with his or her 5 most relevant social anxiety thoughts, and corrective feedback for their particular cognitive errors. The feedback is drawn from a pool of 296 sample answers written for the back end of the program. The user is asked to write 40 different answers to challenge his or her thoughts, using the feedback from the sample answers. In this way, the quality of user responses is shaped across trials.

Module 3: Creating your model

- The user builds his or her own idiosyncratic social anxiety formulation. To prevent errors in constructing the individualized formulation, prewritten symptoms are selected from a list. These include avoided situations, cognitions driving anxiety and avoidance, safety behaviors, mental images, and physical anxiety symptoms. All of the above are presented with detailed education using voice recordings of the clinical psychologists explaining the material.

Module 4: Behavioral experiments

- In this section, the formulation created in the previous section is used to select behavioral experiments to target avoidance and safety behaviors. The user selects an avoided situation from his or her own avoided situations list. The program then creates a behavioral experiment for that situation targeting one or more different cognitions responsible for driving the avoidance and anxiety. The number of experiments to be completed in this section is expected to be around 3-10 for each user. The back end of the program has the potential to create 962 different behavioral experiments for the user. The program determines whether each experiment should be repeated before recommending a novel experiment; this decision is based on whether the user indicates that he or she would still avoid the previously feared situation.

Module 5: Challenge your thinking further

- Advanced cognitive work is presented in this section with a focus on anger. The user is asked to indicate which anger-related beliefs he or she has. The program guides the user through reframing his or her beliefs through a cost-benefit analysis. This is done using material relevant to the user's particular thoughts.

Module 6: Self-processing

- This section targets the maintenance factor of self-focused attention seen in social anxiety. First, to target biased attention in social situations, skills-based attention training [24] is taught to increase the user’s control of attention in social situations. Second, rescripting methods are used to help update faulty and unhelpful imagery [25]. Individualization is particularly important in this section, as the user hears a voice-over that rescripts his or her particular image, selected when the user’s tailored formulation was constructed.

Module 7: Relapse prevention

- This section deals with relapse prevention and reviews all the former components of the program. Furthermore, as depression is a highly comorbid condition in social anxiety, psychoeducation is focused on preventative behaviors that the user can engage in to maintain treatment gains and reduce negative mood.

Postquestionnaires

- The questionnaires that the user responded to at the beginning of the program are administered again. The program creates histograms to show the user his or her scores before and after treatment. The program then creates a PDF document containing all of the program’s materials and the user’s individual data for the user to keep, to help maintain the user’s treatment gains over time.

Our secondary hypothesis was that those who were given access to the program would experience a more positive change in life satisfaction (ie, a greater increase or a lesser decrease) than those who were not. The inclusion of life satisfaction as a secondary outcome reflects a trend toward the development and use of assessments that complement traditional measures of symptom severity by capturing broader changes in psychosocial functioning and quality of life when assessing the impact of interventions [23].

## Methods

### Recruitment

A power analysis indicated that we would require a total sample size of 102 participants (ie, 51 per condition) to achieve a power level of 0.80 at the *P*<.05 level of significance, 1-tailed, assuming a moderate effect size of Cohen *d*=0.5. Our final sample consisted of 65 students (female 47/65, 72%) at a large, public Canadian university, who received credit toward undergraduate psychology courses for their participation. Although some research indicates that social anxiety is more prevalent among women than men (eg, [26]), the unequal sex ratio in our sample is likely attributable, at least in part, to the fact that a majority of students in undergraduate psychology courses at the university at which this research was conducted are female. Recruitment was conducted online, through this university’s psychology department’s “Human Subject Pool” system. The large majority (61/65, 94%) of our participants were first to fourth year undergraduates. Most were either East Asian (31/65, 48%) or white (12/65, 19%), with a mean age of 21.86 years (SD 5.51, range 17-46). All data were collected between September 2016 and June 2017.

All prospective participants completed a prescreening questionnaire online. Participants were deemed eligible for the study if they reported (1) that they were experiencing some degree of social anxiety (see Materials section below), (2) that they had not received treatment for a chronic mental health condition within the 6 months before the commencement of their participation, and (3) that they had not formerly been diagnosed with social anxiety by a clinician. The resulting sample was thus one of students who experienced symptoms of social anxiety but otherwise reported good psychological health at the time of their recruitment. We chose to employ a nonclinical sample for this study to (1) provide a rigorous initial test of Overcome Social Anxiety in a population of participants whom we expected to be less vulnerable than those with clinically diagnosed anxiety, before expanding our testing to a clinical sample, and (2) explore the potential utility of Overcome Social Anxiety in a broad, diverse user base for whom the use of digital technologies is normative and frequent. The full study protocol was approved by the local institutional ethics board.

### Materials

Our materials included an eligibility questionnaire, a baseline questionnaire, a follow-up questionnaire, and Overcome Social Anxiety, the program whose effectiveness this study was designed to assess.

The eligibility questionnaire included the 3-item mini-social phobia inventory (Mini-SPIN) [27], as well as 4 social anxiety assessment items from the *Diagnostic and Statistical Manual of Mental Disorders* (5th edition; DSM-5) [28], all of which were selected to assess prospective participants’ reliance on “safety behaviors” to quell their social anxiety symptoms. Prospective participants responded (1=not at all, 2=a little bit, 3=somewhat, 4=very much, 5=extremely) to each of the 3 Mini-SPIN items (eg, “I avoid activities in which I am the center of attention”) and the 4 DSM-5 items (eg, “I spend a lot of time preparing what to say or how to act in social situations”). Internal consistency for this 7-item scale was good in our sample (Cronbach alpha=.88). Those who endorsed at least 1 of these 7 items with very much or extremely were considered eligible to participate. This criterion was recommended by the third author, who is a professional clinical psychologist. The eligibility questionnaire was also used to screen out those who had formerly been diagnosed with social anxiety disorder by a clinician or had received treatment for a chronic mental health condition within the past 6 months.

The baseline questionnaire consisted of a demographics section, the social interaction anxiety scale (SIAS) [29], the fear of negative evaluation scale (FNE) [30], and the quality of life enjoyment and satisfaction questionnaire—short form (Q-LES-Q-SF) [31]. The multigroup ethnic identity measure—revised [32] was also included in the baseline questionnaire to examine separate research questions about ethnic identity and social anxiety. The follow-up questionnaire consisted of the SIAS, FNE, and Q-LES-Q-SF, with an additional section for treatment condition participants to provide feedback on their experiences using Overcome Social Anxiety.

The FNE is a widely used measure of social anxiety. It consists of 30 binary-choice items, each of which yields a score of 0 or 1, depending on how it is answered, for a total score ranging from 0 to 30. The scale was found to be valid at the time of its initial publication [30], and subsequent research has confirmed that the FNE converges with other measures of social anxiety [33], discriminates between social anxiety and other anxiety disorders [34], and distinguishes between those who exhibit psychological processes characteristic of social anxiety and those who do not [35].

The SIAS measures social anxiety as well. The total of scores for each of its 20 items (0=not at all, 1=slightly, 2=moderately, 3=very, 4=extremely) are summed for a total score between 0 and 80, inclusive. This measure’s validity and reliability have been demonstrated [29,36].

Finally, the Q-LES-Q-SF is a measure of life satisfaction. Although it includes 16 items, the last 2 are stand-alone items; therefore, the final score is derived from the sum of responses to the first 14 items (1=very poor, 2=poor, 3=fair, 4=good, 5=very good), each of which addresses satisfaction in a different domain of life (eg, economic status, social relationships). Research has found the Q-LES-Q-SF to be reliable, valid [31], and useful among diverse populations [37].

### Procedure

Before recruitment, each participant number (ie, first participant, second participant) was randomly assigned, without stratification, to the treatment or wait-list control condition. This predetermination of condition allowed the attending research assistant to prepare the appropriate study materials in advance of each participant’s scheduled lab visit. During their baseline lab visits, those assigned to the control condition were given a brief explanation of the general purpose of the study and, after giving their consent to participate, were asked to complete the baseline questionnaire. Those assigned to the treatment condition completed these same procedures, but were also given a verbal overview of Overcome Social Anxiety, were left alone to browse the program’s website for 8 min, and were set up with an Overcome Social Anxiety account by the attending research assistant.

During the 4-month interval between participants’ baseline and follow-up lab visits, participants in the treatment condition were sent reminders to continue using the program and information about requirements for the reception of course credit 1 month before the deadline for the completion of certain modules. Participants were granted course credit incrementally depending on their progress in Overcome Social Anxiety. The maximum amount of course credit a participant could receive was redeemable for a 3% grade increase in each of 2 psychology courses. At their follow-up visits, which were scheduled to occur 4 months after the initial visits, participants were asked to complete the follow-up questionnaire. Those assigned to the control condition were then given access to Overcome Social Anxiety. All participants were debriefed before their participation was terminated.

### Statistical Analysis

The primary dependent variable was the change in the severity of participants’ social anxiety symptoms during the 4 months between their baseline and follow-up lab visits, as measured by the SIAS and the FNE. The secondary dependent variable was the change in participants’ life satisfaction over that period of time, as measured by the Q-LES-Q-SF. For each of these 3 measures, within-subjects *t* tests were employed to determine whether baseline scores differed from follow-up scores for each condition. Additionally, a change score was calculated as the difference between each participant’s baseline and follow-up score for each measure; between-subjects *t* tests were then employed to compare these change scores, for each measure, across the 2 conditions.

In addition to the aforementioned analyses of data from participants who completed both baseline and follow-up assessments, baseline characteristics of those lost to follow-up were compared with those who completed the study using between-subjects *t* tests and chi-square tests. Multiple imputation using SPSS version 24 (IBM Corp., Armonk, NY) [38] was used to produce 5 datasets with individual missing values on the SIAS, FNE, and Q-LES-Q-SF imputed via a series of multiple linear regression models (monotone method) that predicted missing responses on each outcome measure using sociodemographic and the other outcome variables. The 5 imputed datasets were then used to conduct pooled between-subjects *t* test analyses of difference scores on the SIAS, FNE, and Q-LES-Q-SF between treatment and control conditions and the results compared with the initial between-subjects *t* test analyses based on data from complete cases.

## Results

### Retention of Participants

Overall, 264 prospective participants completed the eligibility questionnaire, 173 of whom were deemed eligible to participate. Of the 101 students who participated, 51 were assigned to the treatment condition. Out of the participants assigned to the treatment condition, 1 withdrew from the study before receiving the intervention due to a misunderstanding of the time required to participate in the study. A total of 30 (30/50, 60%) treatment condition participants and 35 (35/50, 70%) wait-list control condition participants returned for their follow-up lab visits. Although we have no data that directly address why a substantial proportion of our participants were lost to follow-up, likely reasons are that participants did not require further research participation credit toward undergraduate psychology courses, and that participants—all of whom were university students—were subject to many competing pressures (academic, social, financial, etc), and completing their participation in our study was not a high priority for them.

**Figure 1:**
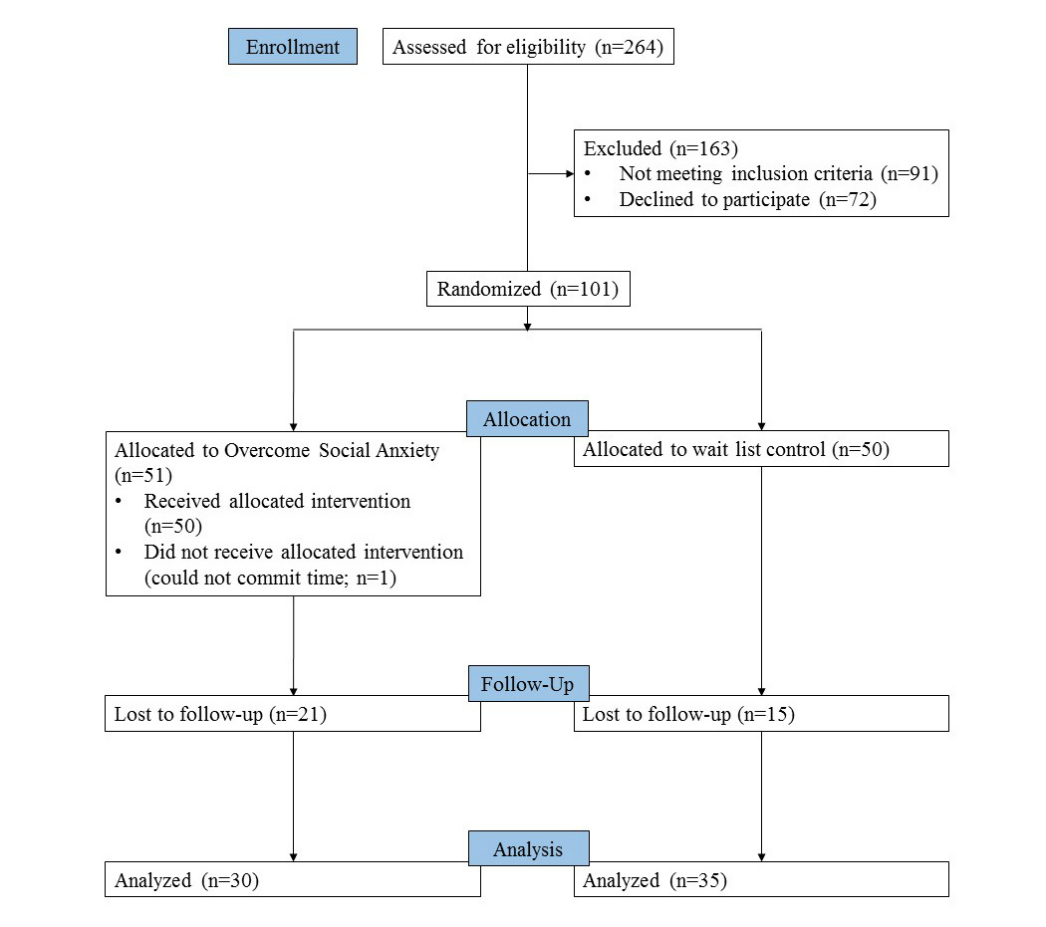
Flowchart of participation.

No significant differences in baseline social anxiety scores (SIAS and FNE), quality of life (Q-LES-Q-SF), age, ethnicity, and gender were found between participants who completed the study and those who did not (all *P*>.05, *t* tests, and chi-square tests). The *P* value for Little test [39] was not significant (χ^2^_4_=3.8 *P*=.43), providing support for the assumption that the missing follow-up data were missing completely at random. The flow of participants through the trial is displayed in Figure 1.

### Baseline Characteristics

The mean SIAS score for the treatment condition was 38.07 (SD 12.75); for the wait-list control condition, the mean SIAS score was 43.60 (SD 13.16). These mean values confirm that our participants had high levels of social anxiety. In comparison, the mean SIAS scores reported by the scale’s creators in a general undergraduate sample, and in a community sample, were 19.0 (SD 10.1, n=482) and 18.8 (SD 11.83, n=315), respectively [29]. Indeed, the SIAS means in our sample even exceeded those in a sample of people diagnosed with social phobia (mean 34.6 [SD 16.4], n=243) [29]. Likewise, mean FNE scores for the treatment (mean 21.40 [SD 6.96]) and control conditions (mean 23.37 [SD 5.53]) in our study were high in comparison with the mean scores of undergraduate samples found by the FNE scale’s originators (mean 15.47 [SD 8.62], n=205) [30] and others (eg, mean 14.26 [SD 7.72], n=539) [35]. Importantly, independent samples *t* tests revealed no significant differences in mean baseline scores between those assigned to the wait-list control condition and the treatment condition on the SIAS (*t*_63_=1.71, *P*=.09), FNE (*t*_63_=1.27, *P*=.21), or Q-LES-Q-SF (*t*_63_=0.35, *P*=.72). Mean questionnaire scores at baseline, among other statistics, are summarized in Table 1.

### Effectiveness of the Intervention

**Figure 2:**
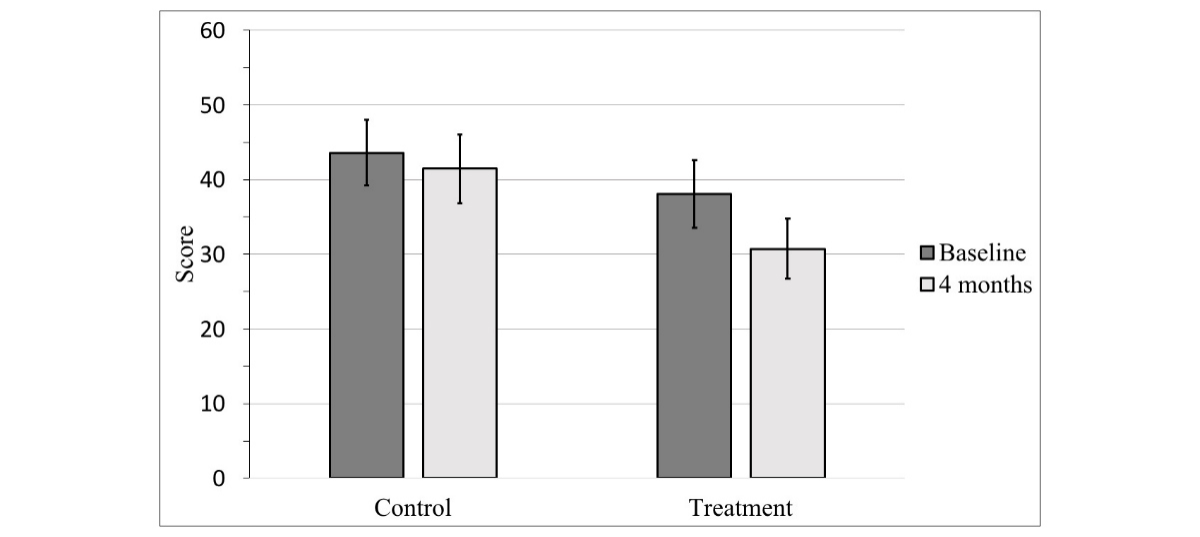
Mean social interaction anxiety scale (SIAS) scores at baseline and 4 months. Error bars represent 95% CIs.

**Figure 3:**
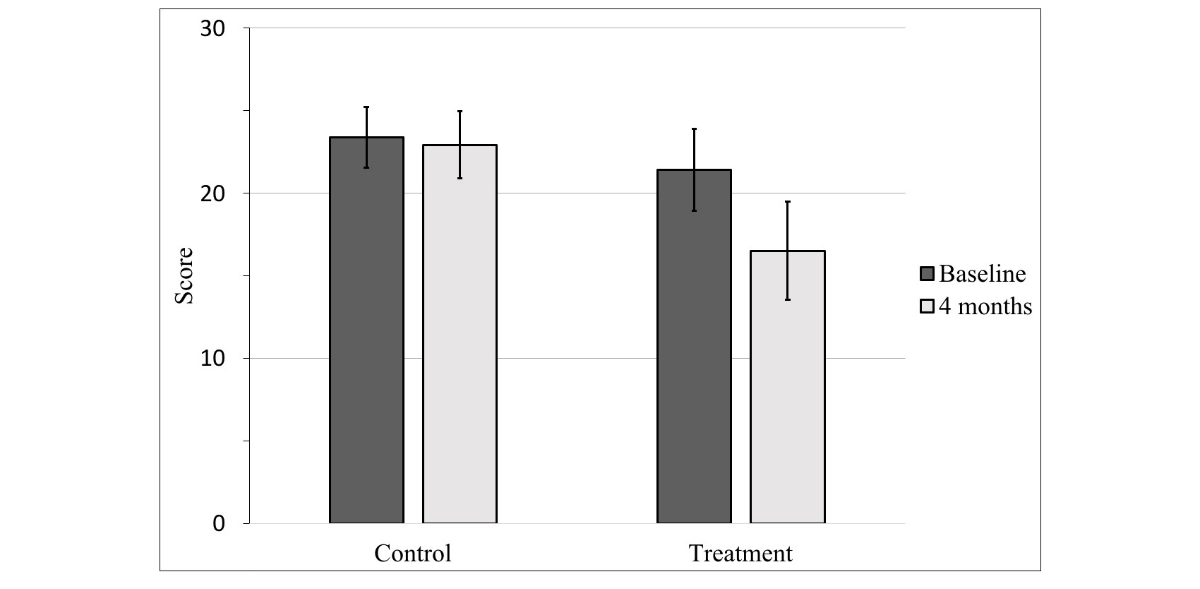
Mean fear of negative evaluation scale (FNE) scores at baseline and 4 months. Error bars represent 95% CI.

Participants assigned to the treatment condition experienced a significant reduction in social anxiety symptoms according to both the SIAS (*t*_29_=3.94, *P*<.001, Cohen *d*=0.72) and the FNE (*t*_29_=4.48, *P*<.001, Cohen *d*=0.82), whereas those assigned to the wait-list control condition did not (SIAS: *t*_34_=1.55, *P*=.13, Cohen *d*=0.26; FNE: *t*_34_=0.85, *P*=.40, Cohen *d*=0.14; see Figures 2 and 3). Neither treatment nor control condition participants experienced a significant change in life satisfaction over the course of the study, as measured by the Q-LES-Q-SF (treatment: *t*_28_=−0.96, *P*=.35, Cohen *d*=−0.18; control: *t*_33_=1.05, *P*=.30, Cohen *d*=0.18). These results are depicted in Table 2. We also compared the 2 conditions with one another directly through an independent-samples *t* test using participants’ change scores (difference between their baseline and follow-up scores).

**Table 1:** Participant characteristics at baseline.

Characteristic		Treatment (n=30)	Control (n=35)	Total (N=65)
Female, n (%)		19 (63)	28 (80)	47 (72)
**Ethnicity**				
	White, n (%)	7 (23)	5 (14)	12 (18)
	Asian, n (%)	19 (63)	21 (70)	40 (62)
	Other, n (%)	4 (13)	9 (26)	13 (20)
Age in years, mean (SD)		21.53 (4.09)	22.14 (6.53)	21.86 (5.50)
Social interaction anxiety scale, mean (SD)		38.07 (12.75)	43.60 (13.16)	41.05 (13.17)
Fear of negative evaluation scale, mean (SD)		21.46 (6.96)	23.37 (5.53)	22.46 (6.26)
Quality of life enjoyment and satisfaction questionnaire—short form, mean (SD)		0.61 (0.13)	0.62 (0.14)	0.61 (0.14)

**Table 2:** Social anxiety symptoms and life satisfaction at baseline and 4-month follow-up.

Condition and time of measurement		Social interaction anxiety scale	Fear of negative evaluation scale	Q-LES-Q-SF^a^
**Treatment (n=30)**				
	Baseline, mean (SD)	38.07 (12.75)	21.40 (6.96)	60.77 (13.32)
	4 months, mean (SD)	30.73 (11.12)	16.50 (8.29)	62.81 (15.53)
	Change^b^	*t*_29_=3.94, *P*<.001, *d*=0.72	*t*_29_=4.48, *P*<.001, *d*=0.82	*t*_28_=−0.96, *P*=.35, *d*=−0.18
**Control (n=35)**				
	Baseline, mean (SD)	43.60 (13.16)	23.37 (5.53)	61.99 (14.17)
	4 months, mean (SD)	41.43 (13.82)	22.91 (6.13)	60.66 (13.77)
	Change^b^	*t*_34_=1.55, *P*=.13, *d*=0.26	*t*_34_=0.85, *P*=.40, *d*=0.14	*t*_33_=1.05, *P*=.30, *d*=0.18

^a^ Q-LES-Q-SF: Quality of life enjoyment and satisfaction questionnaire—short form. In each condition, 1 participant did not complete the Q-LES-Q-SF during the follow-up visits. Data presented in this column for 4-month follow-up and 4-month change are thus based on responses from 29 treatment condition and 34 control condition participants.

^b^This row displays the results of within-subjects *t* tests comparing participants’ baseline scores with their follow-up scores for each measure.

Those assigned to the treatment condition experienced a significantly greater reduction in anxiety than those assigned to the control condition, for both the SIAS (*t*_63_=2.25, *P*=.03, Cohen *d*=0.56) and FNE (equal variances not assumed; *t*_42.54_=3.65, *P*=.001, Cohen *d*=0.97). No significant differences were found between the 2 conditions’ changes in Q-LES-Q-SF scores (*t*_61_=−1.41, *P*=.16, Cohen *d*=0.36).

To assess the potential impact of missing data, multiple imputation was then used to create 5 imputed datasets, and independent-samples *t* tests were used to compare participants’ SIAS, FNE, and Q-LES-Q-SF change scores across treatment and control conditions. Those assigned to the treatment condition experienced a significantly greater reduction in anxiety than those assigned to the control condition, for both the SIAS (*t*_172_=2.12, *P*=.04, Cohen *d*=0.32) and FNE (*t*_110_=3.63, *P*<.001, Cohen *d*=0.69). No significant differences were found between the 2 conditions’ changes in Q-LES-Q-SF scores (*t*_48_=−1.01, *P*=.32, Cohen *d*=0.29).

### Acceptability of the Intervention

Feedback obtained from the 30 treatment condition participants at follow-up was generally positive. Overall, 80% (24/30) of users reported that the quality of support they received from Overcome Social Anxiety was “good” or “excellent,” 87% (26/30) reported “generally” or “definitely” getting the kind of support they wanted from the program, 83% (25/30) responded with “I think so” or “definitely” when asked whether they would recommend Overcome Social Anxiety to a friend in need of similar help, 80% (24/30) reported being “mostly satisfied” or “very satisfied” with the program overall, and 77% (23/30) reported that the program “helped” them or “helped [them] a great deal” to deal more effectively with their problems.

## Discussion

### Principal Findings

The primary dependent variable of this study was change in social anxiety symptoms over a 4-month period. Our hypothesis, namely, that those assigned to the treatment condition would experience a greater reduction in social anxiety than those assigned to the wait-list control condition, was well supported by both the SIAS and FNE results. Indeed, the effect sizes for treatment condition participants’ reduction in social anxiety symptoms over the course of the study, as measured by both the SIAS (Cohen *d*=0.72) and FNE (Cohen *d*=0.82), were approximately *triple* the mean effect size of 6 stand-alone, internet-based CBT treatments for anxiety and depression (Cohen *d*=0.24) found in a meta-analysis [16]. A direct comparison of the treatment and wait-list control conditions’ 4-month change scores on the FNE also revealed a large effect size (Cohen *d*=0.97). In fact, this effect was larger than the mean effect size, calculated between conditions, of 19 randomized controlled trials of computer-aided interventions for anxiety disorders found in a review (mean Cohen *d*=0.96) [40], despite the fact that the interventions in this review all benefited from therapist support. This is surprising, given the clear relationship the authors of this review found between the effect sizes in these 19 studies and the amount of therapist support those studies’ participants received. In other words, even though therapist support appears to contribute substantially to the effectiveness of computer-delivered CBT for anxiety, our findings indicated that Overcome Social Anxiety is comparably effective to therapist-assisted interventions when delivered as a stand-alone treatment.

Our secondary hypothesis was that those assigned to the treatment condition would experience a more positive change in life satisfaction from baseline to follow-up than those assigned to the control condition. Our results did not support this hypothesis, as neither the differences between treatment condition participants’ baseline and follow-up Q-LES-Q-SF scores (*P*=.30, Cohen *d*=−0.18) nor the differences between treatment and control participants’ baseline-to-follow-up change scores on that measure (*P*=.16, Cohen *d*=0.36) were significant. Descriptively, however, participants in the treatment condition showed a small increase in life satisfaction over the 4-month period, whereas participants in the control condition showed a small decrease. It may be that further gains in life satisfaction require additional time, post treatment, to accrue. Alternately, the most clear-cut effects of the intervention may be relatively specific to social anxiety symptoms, with less generalization to life satisfaction.

Finally, participants’ extreme scores on both the SIAS and the FNE suggest that—although we excluded those who had formerly been diagnosed with social anxiety—many of our participants may have met diagnostic criteria for the disorder had they previously received help. To the extent that this is true, it provides an unintended illustration of the severity of social anxiety’s undertreatment among university students.

### Limitations and Future Research

On account of a smaller initial sample size and higher dropout rate than we had anticipated, we failed to reach our target sample size. The lack of significant differences in baseline characteristics between participants who were lost to follow-up and those who completed the study and the results of the multiple imputation support the interpretation of results based on the analyses of complete cases. However, our sample’s unequal sex distribution, generally homogenous age distribution, and unanimously high level of education all limit the generalizability of our findings to other populations. Although not presented in the results, separate subgroup analyses of males and females showed a similar pattern of results to the pooled results, suggesting that the intervention is equally effective in males and females.

In terms of future research, it would be informative to investigate the program’s effectiveness among clinical populations and populations less comfortable with digital technology than the students who participated in our study.

Because the wait-list control condition did not receive any treatment, the treatment condition’s greater reduction in social anxiety symptoms may be attributable, at least in part, to an expectancy effect. It should be noted, however, that because Overcome Social Anxiety is targeted primarily toward those for whom access to human-delivered treatment is limited, any benefit to users—whether attributable to the content of the program itself or attributable to an expectancy effect—is a desirable outcome. Nonetheless, comparing the effectiveness of Overcome Social Anxiety with that of other Web-based and human-delivered treatments would be an important avenue for future research.

Additionally, the research assistants who provided instructions to participants during the follow-up session were aware of participants’ conditions due to prior correspondence and interaction with them. This introduces the possibility of an experimenter effect in our outcome measures, although we believe that any such effect would have been mitigated by the fact that research assistants were positioned such that they were unable to see the computer screen on which participants completed questionnaires. However, future research evaluating the effectiveness of Overcome Social Anxiety would benefit from blinding those who administer outcome measures to each participant’s condition.

Interestingly, the fact that participation in this study was extrinsically motivated through our granting of course credit may have affected our findings in 2 opposing ways. On one hand, it is reasonable to suppose that the program may have been less effective among our extrinsically motivated participants than it would be among the program’s intended users, whom we presume to have predominantly intrinsic motivations for using it; for example, extrinsically motivated users may simply put less effort than intrinsically motivated users into maximizing their benefit from the program. On the other hand, our incremental granting of course credit—which was contingent upon the number of modules each treatment condition participant completed—may have artificially inflated adherence in our sample. Thus, participants in our sample may have benefited less from their usage of the program, per hour of use or per module completed, but spent more hours and completed more modules overall than the typical intended user.

Although our findings provide preliminary evidence that Overcome Social Anxiety is effective, further research will be required to elucidate which elements of the program contributed most to its effectiveness. Future research comparing several variants of Web-based interventions could prove particularly fruitful in discerning which aspects of a Web-based intervention are most important to its ultimate success.

### Conclusions

Our findings show that Overcome Social Anxiety is an effective program for reducing social anxiety symptoms. Additionally, the mechanisms it employs to overcome the limitations of previous Web-based CBT interventions appear to have been successful and may thus help guide the development of future Web-based treatments. Finally, our results indicate that developments at the intersection of psychology and technology are now sufficient to create effective, stand-alone, computer-delivered therapy programs, highlighting the opportunity for further research in this exciting area.

## Appendix

Multimedia Appendix 1 Screenshot of Overcome Social Anxiety.

Multimedia Appendix 2 CONSORT-EHEALTH checklist (V 1.6.1).

## Abbreviations

CBT: cognitive behavioral therapy
DSM-5: Diagnostic and Statistical Manual of Mental Disorders, 5th edition
FNE: fear of negative evaluation scale
Mini-SPIN: mini-social phobia inventory
Q-LES-Q-SF: quality of life enjoyment and satisfaction questionnaire—short form
SIAS: social interaction anxiety scale
