# Supplementary figures and images for: Evaluating a Web-Based Social Anxiety Intervention Among University Students: Randomized Controlled Trial
Source: J Med Internet Res. 2018 Mar 21;20(3):e91. doi: 10.2196/jmir.8630 (PMC5885061; doi:10.2196/jmir.8630)

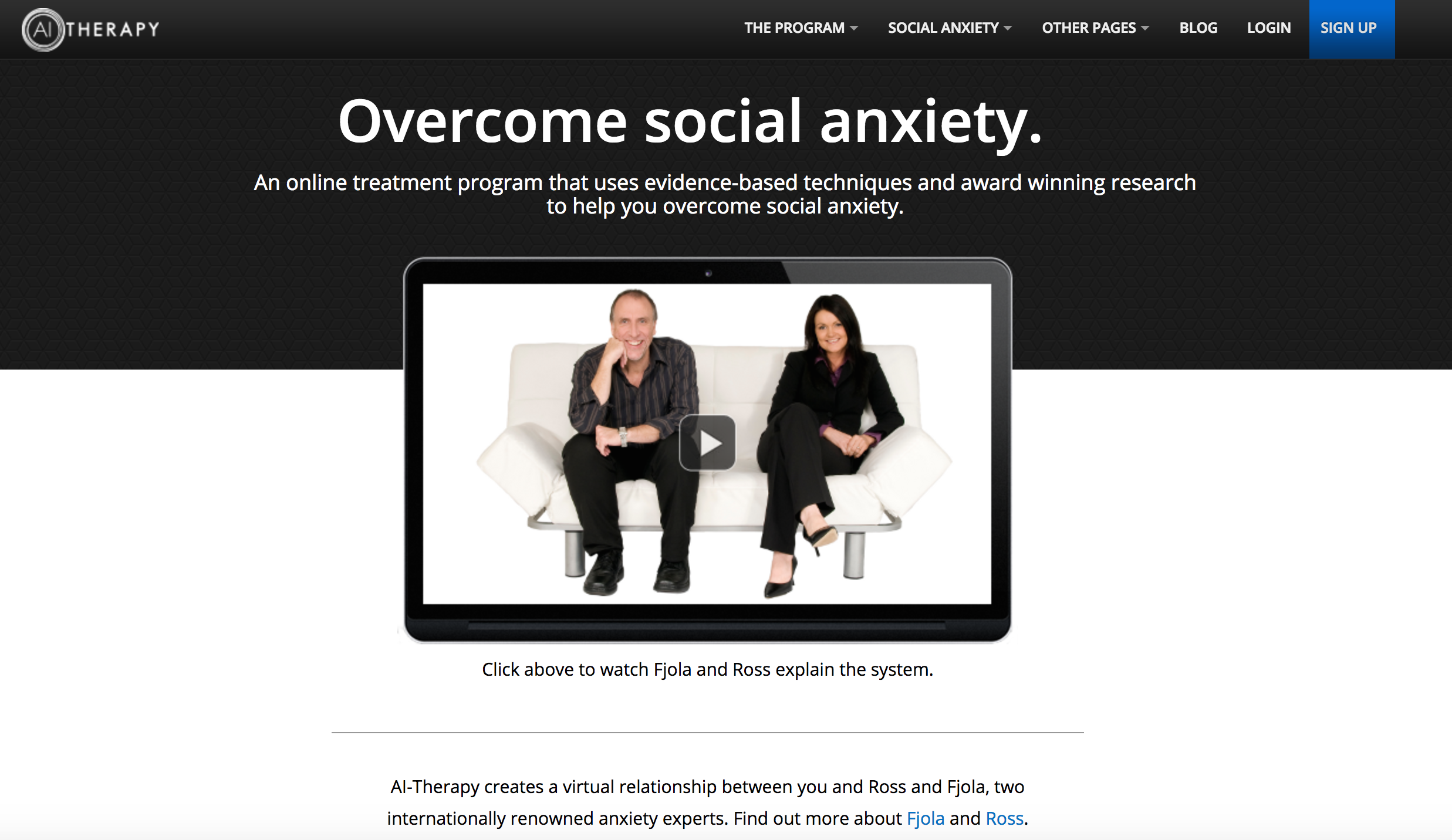

Supplement: Multimedia Appendix 1 [file jmir_v20i3e91_app1.png]
